# Supplementary material for: Competitive processes shape multi-synapse plasticity along dendritic segments
Source: Nat Commun. 2024 Aug 31;15:7572. doi: 10.1038/s41467-024-51919-0 (PMC11365941; doi:10.1038/s41467-024-51919-0)
Supplement: Supplementary file 1 — Supplementary Information [file 41467_2024_51919_MOESM1_ESM.pdf]

Supporting information

ROI detection algorithm

To identify a spine ROI, we (i) determined the dendrite medial axis and (ii) manually selected the spine centre. To identify the dendrite position, we marked on the beginning and end of the dendrite stretch of interest, and a breadth-first search[81] identified the dendritic path. A putative spine centre was determined to calculate the spine ROI by manually selecting the centre coordinate. Once the spine centre was obtained, the ROI was calculated by stepping outward, pixel by pixel, on eight rays that formed an irregular octagon. Next, our algorithm checked if a set of rules were met at each step, and if any of them were broken, a counter was increased proportionally at the corresponding point. These rules are as follows

1. **Boundary Rule**
   If the ROI ray exceeds the boundary of the image, this ends the progression in that direction.

2. **Contour Rule**
   The image is treated with a Canny edge detection algorithm which calculates edges in an image. Depending on the distance from x_0, encountering such an edge counts as a certain number of strikes.

3. **Normalised fluorescence fall-off Rule**
   Suppose the normalised fluorescence of the test point becomes one-third of the initial normalised fluorescence of x_0, or the normalised fluorescence falls below four times the background. In that case, the algorithm counts this rule as broken.

4. **Dendrite Rule**
   We assume that the spine is on average symmetrical and that the user will select the centre of the spine. Therefore, assuming that the initial point x_0 is outside the dendrite, this rule is broken once the test point is closer to the centre of the dendrite than to x_0.

5. **Normalised fluorescence increase Rule**
   Suppose the normalised fluorescence increases on consecutive steps away from x_0. In that case, we have most probably entered the dendrite, and so after a certain amount of steps, we also consider this a rule break.

Once all rays have stopped, the ROI is drawn by connecting the eight points into a shape encompassing the spine. This octagonal ROI is then used to determine the normalised fluorescence quantities. To determine the interior of the ROI, we use a simple test involving the winding number algorithm[82], which sums up the angles subtended by each side of the polygon. If this number is non-zero, the test point is inside the polygon. A full description of the underlying algorithms and image analysis pipeline can be seen in[76].

Details on the parameter fitting algorithm

Here, we present the equations that drive the gradient-based adjoint approach[83] described in the model analysis section but will omit the derivation thereof as it lies outside the scope of this article. Direct solutions of the gradients are evaluated numerically to acquire the required parameter updates. The first three equations define the adjoint state variables (denoted by the $(\cdot)^\dagger$) that deliver the sensitivity information of the model to changes in the parameters. The parameters without the dagger remain the same as those observed in the model equations. Finally, L_i was introduced in the numerical methods section and denotes the averaged spine

$$\frac{\partial C^\dagger}{\partial t} = -\alpha_1 \frac{\partial^2 C^\dagger}{\partial x^2} + \alpha_2 C^\dagger + \beta_2 P_{\text{in}}({P_{\text{in}}}^\dagger - P^\dagger) - S^\dagger \tag{14}$$

$$\frac{\partial {P_{\text{in}}}^\dagger}{\partial t} = -\beta_1 \frac{\partial^2 {P_{\text{in}}}^\dagger}{\partial x^2} + \beta_2 C({P_{\text{in}}}^\dagger - P^\dagger) \tag{15}$$

$$\frac{\partial P^\dagger}{\partial t} = -\beta_1 \frac{\partial^2 P^\dagger}{\partial x^2} + \gamma P^\dagger - \zeta F(S) S^\dagger \tag{16}$$

$$\frac{\partial S^\dagger}{\partial t} = -\zeta S^\dagger P F'(S) + \sum_i 2(S - L_i) \tag{17}$$

These equations denote the required calculations involving both the model and adjoint variables to calculate the gradient that will lead to better fits. These variables then provide us gradient information we can use to update our parameter estimates.

We emphasise that this fitting was only used in the initial analysis of the 3 spine and to alter the ratios of ζ_1 and ζ_2 in the drug condition experiments. For all other experiments, model predictions were used.

Local versus global effects

On the spatial and temporal scales investigated, we have found that the spines sharing a short region of dendrite appeared to be competing for a limited resource within a dendrite. However, this result does not rule out changes that may cause additional competition occurring elsewhere in the neuron or extracellularly, outside the imaging ROI, nor can it account for plasticity events that may act to rebalance the network on longer timescales than explored here. Presumably, the clustered potentiation induced in these experiments must have upper limits (as demonstrated in the 15-spine stimulation condition), and homeostatic mechanisms that normalise neuronal activity levels (such as synaptic scaling of AMPARs) may operate over slower timescales than the time course of spine changes monitored in the current study. Following these same dendrites over longer timescales (hours to days) may reveal additional patterns of synaptic plasticity that are not apparent here.

The stimulation paradigm we have considered here does not involve a combination of presynaptic and postsynaptic stimulation and, therefore, in principle, it is non-Hebbian; direct uncaging of glutamate onto postsynaptic spines has been used to induce sLTP directly. We find that a single stimulation induces a small amount of sLTP, whereas when 3, 7, or 15 spines are stimulated, dynamics which can be considered consistent with a Hebbian learning component occur (presumably via increased membrane voltage at the postsynaptic dendritic segment, which is shared by all synapses). This is in line with previous work, such as[22], reporting that local glutamate uncaging with cell-wide depolarisation and, for groups of 7 spines, homosynaptic inputs lead to robust sLTP. Building on our findings and combining presynaptic and postsynaptic stimulation in future sLTP/sLTD experiments could help dissect the interaction of Hebbian and non-Hebbian components across time and dendritic space, for instance, by stimulating a controlled number of spines by varying the timing of the glutamate uncaging relative to the timing of a depolarising current into the dendrite[84].

Additionally, regarding the sum total growth of estimated spine volumes we have observed, we note that we cannot exclude the local synthesis of proteins. The results obtained within the studied spatial and temporal scales are consistent with other studies. This effect may require additional consideration in revising the model for a future study as the total stores shared among spines may not be constant in time or space, as is assumed here.

Presynaptic identity

Our current study focused entirely on monitoring dendritic spine strength using the intensity of a fluorescent protein (GFP) as a readout. This is a well-used and validated approach, both *in vitro* and *in vivo*, and for example, has been used to demonstrate sLTP associated with behavioural task acquisition[33,85]. Single-cell expression of the fluorophore allows excellent signal-to-noise readout of GFP spine signals and the ease of imaging large populations of inputs over time. However, the method does not give information about the presynaptic identity of the axons arriving onto the spines. Our study used apical oblique dendrites of CA1 neurons, and thus, the incoming axons were likely part of the Schaffer collaterals originating in CA3[86]. These inputs have been shown to make multiple contacts onto single CA1 neurons[10], including onto single dendritic branches.

It would be of interest to determine the plasticity rules when a cluster of dendritic spines sharing the same presynaptic input is potentiated and how well such rules are matched to spine behaviour following plasticity induction with synchronous glutamate uncaging that bypasses communication with the presynaptic terminals. In our study, glutamate uncaging of smaller cluster sizes (3 and 7 spines) could reflect a single or a pair of inputs being potentiated together. We note that a related study[74] showed that same-dendrite same-axon pairs are rare in the middle of stratum radiatum and, hence, the presynaptic axon could not be conclusively linked to cluster coordination. In contrast, these same-dendrite same-axon pairs were more common in the distal tufts of CA1 and might lead to fruitful future study[87]. Further work is required to determine if individual inputs are recognised and privileged from a postsynaptic point of view.

Biological candidates for P and C

Up to this point, we have not speculated on the exact nature of the C and P. However, several candidates have been previously studied that might represent these quantities. We stress that Cs and P may not necessarily be restricted to single molecules but may represent a group of molecules that act collectively to achieve the results seen in the experiments.

In our experiments, we have presented results obtained when inhibiting calcineurin and CaMKII, which indicate that a possible candidate for P is CaMKII. Previous studies have shown that CamKII, activated by an increase in Ca^{2+}, is critical for sLTP[88,89]. Moreover, active CaMKII can be converted into a Ca^{2+}-independent, persistently active state via autophosphorylation[90]. Experimental and theoretical evidence suggests that the concentration of active CaMKII evolves on the time scale of minutes[91], which is much slower than the Ca^{2+}-mediated dynamics

lasting a few seconds that has been considered by previous models[38,39]. Additionally, recent studies have reported a role for CaMKII in LTD also[92,93], such that CaMKII could potentially serve as a dual-role plasticity molecule represented by P.

Calcineurin inhibition has previously been seen to prevent heterosynaptic sLTD without affecting homosynaptic sLTP within a spatial extent of 4μm of the activation site[21,22], whilst inhibition of CaMKII leads to deficits in sLTP[30] and heterosynaptic sLTP[22]. Other candidate molecules for P include Inositol trisphosphate receptors (IP3Rs) which have a similar effect on sLTP as calcineurin[21], h-Ras which has been seen to lower the threshold for neighbouring spine sLTP induction[14] or RhoA which, following synaptic activity, exits the spine and diffuses along the dendrite[26]. In Fig. S2 we show that the diffusion coefficients of the possible biological correlates of P are consistent with the range of diffusion coefficients we obtained for P. Additionally, it is conceivable that our model variable P represents the combined action of multiple molecules. In this case a mix of the different CamKII proteins could also be potential biological correlate of P.

Candidates for C include Ca^{2+} which is a key factor for synaptic plasticity[37], the ARC gene product, which acts in the form of slow homosynaptic sLTP followed by heterosynaptic sLTD[50] or NO synthase that produces nitric oxide, which in turn facilitates sLTP and affects synaptic strengths of neighbouring neurons[94,95]. We emphasise that our model does not rely on a particular molecular identity of the parameter C; instead, we believe it exhibits the characteristics consistent with the above mentioned candidate proteins/molecules. Therefore, C can be interpreted to be a protein, a small molecular compound that reacts quickly to spine stimulation, or potentially a combination of both.

Designing experiments with these quantities in mind could reveal more about the nature and composition of both the C and P.

Calcium imaging

To obtain the results of Fig. S3 neurons were biolistically transfected with GCaMP6s and DsRed, and used for experiments 48-72 hours later. Both GCaMP6s and DsRed were excited at 910 nm. Uncaging conditions were identical to the other experiments in the manuscript (i.e. 60 pulses at 1 Hz, pulse width 4 msec), except dendrites were simultaneously imaged at 1 Hz throughout the stimulus, including short (5 second) baseline and chase periods. To measure spine and dendritic calcium dynamics during glutamate uncaging, ROIs were positioned over the targeted spines, and the dendrite directly below the targeted spine. For clusters of spines, the dendritic ROI was positioned in the centre of the cluster. GCaMP6s intensity was measured using Fiji[96]. To measure dendritic spatial calcium dynamics, a line profile was drawn along the dendrite and expanded to fill the internal width of the dendrite. This was used to measure both the GCaMP and the DsRed signal intensity along the dendrite. The GCaMP signal was normalised to the DsRed signal for the entire recording, then the average during the stimulus was calculated (excluding baseline and chase period), and this was normalised to the baseline. This was then binned to reduce noise. All individual dendritic profiles were then aligned to the stimulated spine, or in the case of a cluster, the centre of the cluster.

Full model parameters and equations

For completeness sake, we include here the full set of model equations as well as their corresponding initial conditions at the time of stimulation (i.e., during baseline: defined by $t_{\text{stim}} = 0$) are listed below:

$$\frac{\partial C}{\partial t} = \alpha_1 \frac{\partial^2 C}{\partial x^2} - \alpha_2 C, \tag{18}$$

$$\frac{\partial P_{\text{in}}}{\partial t} = \beta_1 \frac{\partial^2 P_{\text{in}}}{\partial x^2} - \beta_2 P_{\text{in}} C, \tag{19}$$

$$\frac{\partial P}{\partial t} = \beta_1 \frac{\partial^2 P}{\partial x^2} + \beta_2 P_{\text{in}} C - \gamma P, \tag{20}$$

$$\frac{\partial S}{\partial t} = \zeta_1 C + \zeta_2 P F(S), \tag{21}$$

where the decision variable of P, $F(S)$, is defined by:

$$F(S) = -\tanh(\phi(S - \nu)). \tag{22}$$

Parameter	Role	Equations
C_s	Initial amount of C present in each spine (synaptic store of C)	(1)
C_d	Initial amount of C present in the dendrite local to the stimulated spine (dendritic store of C)	(1)
λ	Effect of distance of other stimulations on competition	(3)
α_1	Diffusion rate of C	(4)
α_2	Degradation rate of C	(4)
ρ	Initial amount of P_{in} throughout the dendrites and spines	(5)
β_1	Diffusion rate of P_{in} and P	(6), (7)
β_2	Translation rate of P_{in} into P	(6), (7)
γ	Degradation rate of P	(7)
ϕ	Parameter that defines how fast P flips from potentiating to depressing	(8)
ν	Location of the flip of P from potentiating to depressing	(8)
ζ_1	Strength of C on the change of the spine size	(9)
ζ_2	Strength of P on the change of the spine size	(9)

Table S1: Model parameters, their biological role and relevant model equations

Additionally, the initial conditions for C and P_{in} are defined as

$$C(x,0) = \sum_{i=1}^{N} (C_s + C_d D_i) \exp(-1000(x - x_i)^2), \tag{23}$$

$$P_{\text{in}}(x,0) = \rho \left(1 - \sum_{i=1}^{N} (1 - D_i) \exp(g(x - x_i)^2\right). \tag{24}$$

where the competition function D_i, is defined by

$$D_i = \frac{1 + d_i}{N + \hat{N}}, \tag{25}$$

and

$$d_i = \sum_{j=1, j\neq i}^{N} \left(\frac{|x_i - x_j|}{1 + |x_i - x_j|}\right)^{\lambda}, \tag{26}$$

In total, our model has 13 distinct parameters to be determined experimentally. They are listed, with their biological role in table S1. Let us note that the effects of certain biological parameters, such as the degradation rates or location of the P threshold, can be intuitive to understand. However, interpreting the effects of other model parameters such as the steepness of the function $\phi(S)$ or the ratio C_s/C_d may be harder. To build intuition, we studied the effects these parameters have on the plasticity outcomes. Removing the spine-specific component of C_s we observed less competition as more spines are stimulated and the shared dendritic store is depleted. On the other hand, when we removed the competitive element of C (C_d) this resulted in stronger potentiation among stimulated spines since more C was now activated per stimulation site. Let us note that the exact C_s/C_d ratio may be hard to pin down precisely because of our temporal resolution in the data, but each value, C_s and C_d, seems to be important for the ability of our model predictions to be valid across different stimulation scenarios. In addition, the C_s/C_d ratio had an impact on the difference between edge and middle spines. In stimulation scenarios where the spine competition is driven primarily by the fast C dynamics, our model predicts a rapid differentiation between edge and middle spines, whereas if the spine competition is driven primarily by the P dynamics (which acts on slower scales), the model predicted edge-to-middle difference later, after 20 minutes.

Studying the effect of ϕ on the plasticity outcome we found that it mediates how strongly P is able to contribute to potentiation and whether its contribution is of equal strength as that of C. For example, when using the smaller phi value, $F(S)$ exhibits a shallow slope and the potentiation effect of P will be severely dampened close to the threshold. In contrast, using a large ϕ will lead to an instantaneous shift from potentiation to depression. Similarly, if ϕ is small, the size-dependent feedback mechanism is not able to suppress growth of large spines which means that potentiation amplitude will be stronger for small ϕ.

Implementing temporal scales in the stimulation paradigm

Our current model is only equipped to handle co-current stimulations, i.e., the stimulations are assumed to be simultaneous. By modifying the distance function d_i (see eq. (3)), we can obtain a combined spatial-temporal distance function, $\hat{d}_i$, which similarly measures the temporal difference between stimulations as

$$\hat{d}_i = \sum_{j=1, j \neq i}^{N} \left(\frac{|x_i - x_j| + |t_i - t_j|}{1 + |x_i - x_j| + |t_i - t_j|} \right)^{\lambda} . \tag{27}$$

where t_i is the timepoint of the ith stimulation and μ defines the strength of the temporal effect. We combine spatial and temporal information so that competition only occurs when they are close in space and time. With $\hat{d}_i$ in hand, we can substitute it into eq. (2) to generate the initial conditions for C and P_{in}.

By utilising this form, we mirror the model predictions related to the spatial information, i.e., events far apart in time will decouple while events close in time will lead to competition for resources. Currently, our experiments do not have this temporal information. However, future work will explore this avenue to confirm whether this symmetry relation between spatial and temporal is indeed the correct form.

Supporting Figures

Heterosynaptic effects

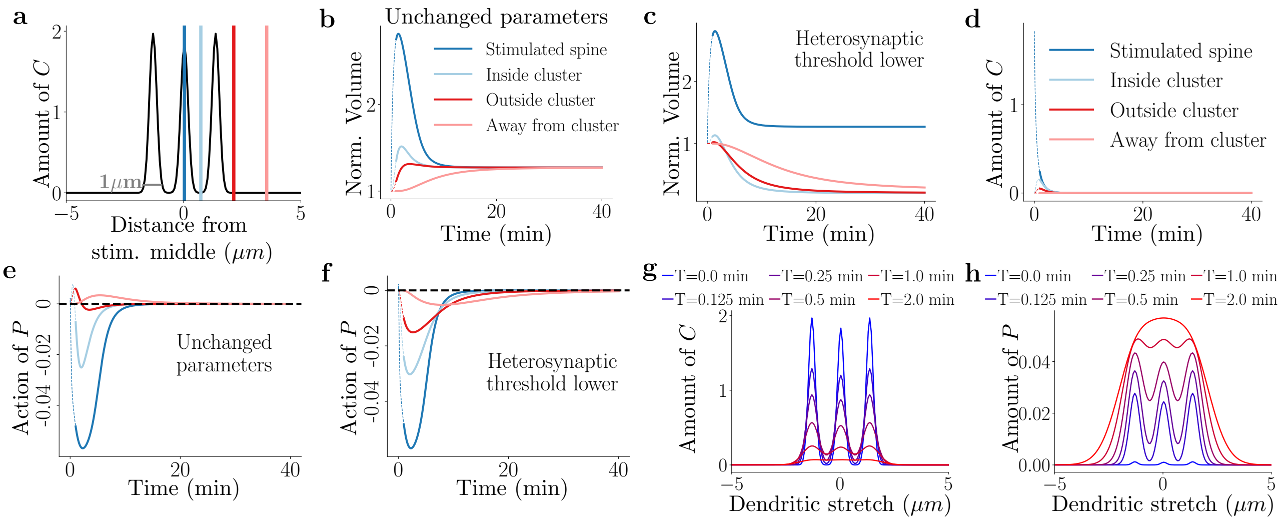

Figure S1: **By studying locations in the spatial dimension, the model can make predictions for spines that are not directly stimulated (heterosynaptic) a)** Spatial arrangement of the initial C distribution for three simultaneous stimulations. Vertical lines represent sample locations where we study S, P and C dynamics (b-f). The grey horizontal line depicts the approximate size of the Gaussian initial condition in terms of μm. **b)** The temporal dynamics of the spine locations of a) (with the same colour code) and the parameters of the original model in Fig. 2. We observe the clear potentiation of the stimulated spine (dark blue) and mirrored, but lower response of the heterosynaptic spine inside the cluster (light blue). The dynamics of the spines outside the cluster (dark and light red) show dynamics that exhibit long-term characteristics. Additionally, the temporal effect of the spine furthest from the cluster growing only as resources diffuse to it, is also observed. **c)** By implementing a lower P threshold in the model (see equation (8)) specifically for the heterosynaptic spines, sLTD is observed in those spines (in line with experimental results such as[21]). **d)** Dynamics of C plots at the selected locations demonstrate that C exhibits fast dynamics that do not affect sites further away from the cluster. These dynamics are the same both for the original parameter set and the lowered parameter set, as the threshold only affects the action of P. **e)** The action of P of the original model parameters demonstrates long-time dynamics. Additionally, for the spine away from the cluster (light red), the growing plasticity response is primarily driven by P. **f)** When considering the P dynamics of the lowered-threshold model, P only acts in a depressing manner for all spines. **g-h)** Given the 3 spine model simulation, we can gain insight into the spatial dynamics of the C and P components in (g) and (h), respectively. We note that the C dynamics are highly localised and dominated by the degradation term, while P primarily diffuses, thus being found at locations further from the stimulation site as well as accumulating around the stimulation sites.

Experimentally reported protein diffusion coefficients

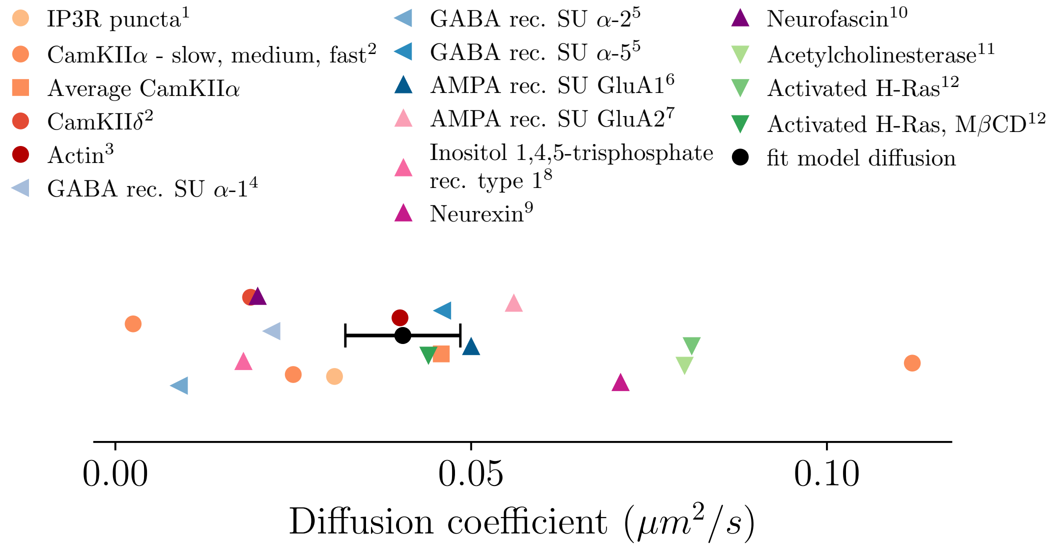

Figure S2: The diffusion coefficient we obtained in our model fit of the 3-spine stimulation experiment (black dot with error bars) is consistent with many experimentally observed diffusion coefficients of possible biological candidates for the component P. Abbreviations rec. and SU stand for receptor and sub-unit, respectively. [1]Thillaiappan *et al.* [2017], [2]Datapoints refer to a slow, medium-fast and fast CamKIIα population as well as CamKIIδ populations from Lu *et al.* [2014], [3]Hannezo *et al.* [2015], [4]Muir & Kittler [2014], [5]Hausrat *et al.* [2015], [6]Mikasova *et al.* [2012], [7]Renner *et al.* [2012], [8]Pantazaka & Taylor [2011], [9]Neupert *et al.* [2015],[10]Boiko *et al.* [2007],[11]Peng *et al.* [1989],[12]Murakoshi *et al.* [2004].

GCaMP6s in spines and dendrites during uncaging

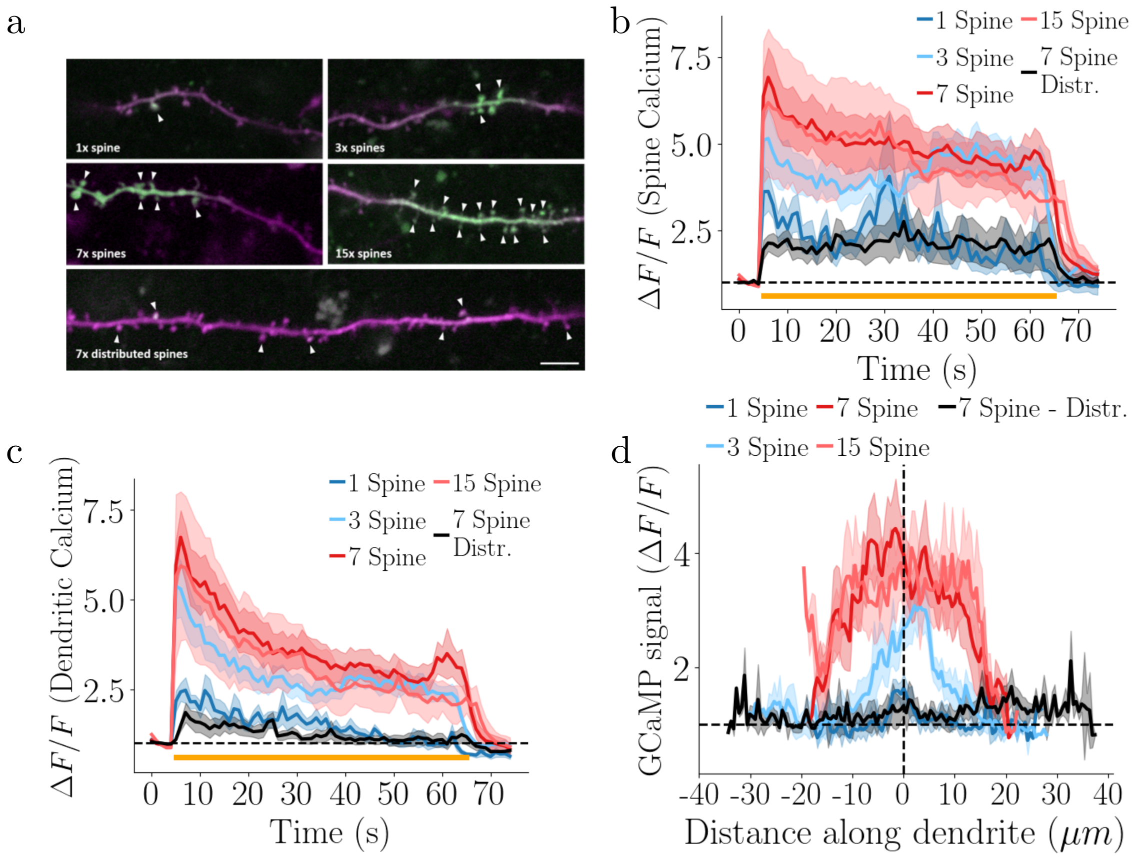

Figure S3: **Imaging of GCaMP6s in spines and dendrites during uncaging. Dendrites from neurons expressing GCaMP6s and DsRed were targeted for simultaneous imaging and glutamate uncaging. a)** Example images illustrating spatial spread of calcium during uncaging. Green, GCaMP6s, magenta, DsRed. Scale bar = 5 μm. **b)** Spine and **c)** dendritic calcium dynamics during uncaging. Repetitive glutamate uncaging (yellow bar) elicits a sustained spine and dendritic calcium elevation during stimulation, which returns to baseline levels within seconds of the cessation of uncaging. Measuring calcium elevations along the dendritic shaft demonstrates that calcium rises are spatially confined close to targeted spines, and that peak calcium occurs in the centre of the stimulated spine cluster. **d)** Measured average spatial profile of calcium during uncaging stimulus. All individual experiments were aligned to the stimulus spine (for the 1x spine condition) or to the centre of the stimulated cluster (for the 3x, 7x, 15x, and the distributed 7x conditions). N's (cells); 1x = 8, 3x = 9, 7x = 11, 15x = 4, 7x distributed = 7.

Specificity of spine stimulation

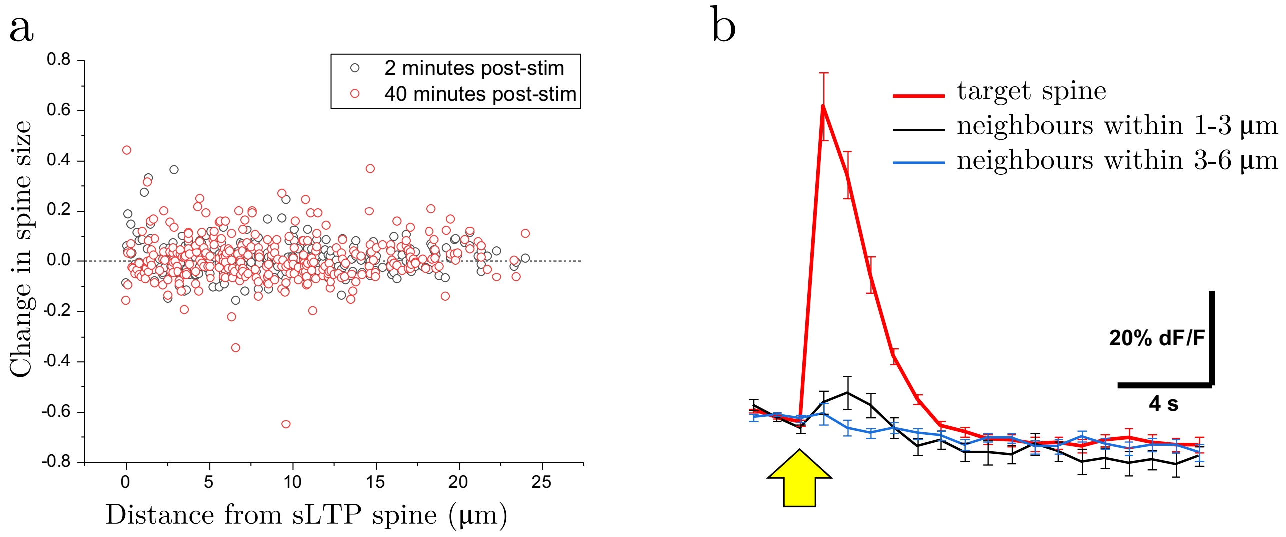

Figure S4: **Single-spine stimulation is specific to the targeted spine. a)** Changes in neighbouring spine size following sLTP induction of a single target spine. Change in heterosynaptic, non-stimulated spine size is plotted against distance from stimulated spine sharing the same dendrite. (Data from 10 neurons, 299 neighbouring spines). **b)** Calcium responses in targeted spines and neighbouring spines. Single 1 msec pulses of glutamate were delivered 0.5 μm from a target spine head (yellow arrow), and GCaMP6s fluorescence was measured in it and nearby neighbouring spines in the same z imaging plane. Calcium transients are largely restricted to the target spine. (Data from 3 neurons, 10 target spines, 29 neighbouring spines).

Edge-to-middle difference as a function of cluster size

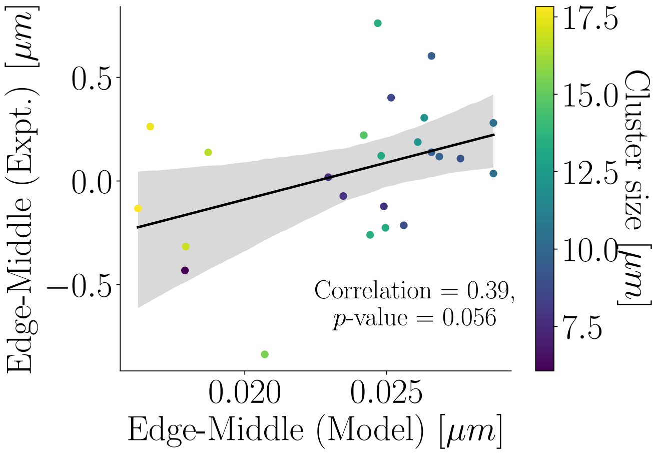

Figure S5: **Predicted vs measured edge-middle difference.** Y-axis shows the edge - middle spine plasticity difference in the experiment and y-axis the corresponding data for the model alongside a linear regression (with a 95% confidence interval) and the calculated Pearson correlation coefficient. This analysis indicates that there is a measure of positive correlation (+0.39), at a p-level of 0.056. N = 24 clusters.

Raw spine size distributions

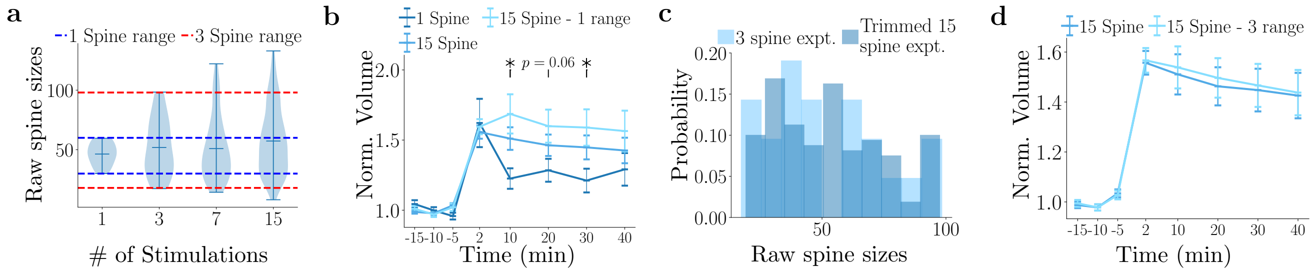

Figure S6: **Co-stimulation of multiple spines changes the plasticity response of initially similarly sized spines.** **a)** Raw spine size distributions across 1-, 3-, 7- and 15 spine stimultion experiments. The percentage of spines in the 7 and 15 spine experiments whose sizes fall in the range of the 3 spine experiment is 86.7% percent and 88.3% percent, respectively. **b)** By considering only the spines in the 15-spines experiments that fall within the 1-spine experiment (blue dotted line in a), we compared 1-spine and 15-spine dynamics that consisted of spines with similar initial sizes. Despite being in the same initial size range, the dynamics of the 15 spine - 1 range line (lightest blue) shares the dynamics of the full 15 spine example and does not show a significant difference, whereas when compared to the single spine dynamics, it significantly differs at +10 min and +30 min time points (p = 0.022, 0.037, respectively. t-test with multiple pairwise correction). **c)** Similarly, we can constrict the 15 spine experiment to the 3 spine range (red dashed line in a). Statistical tests confirmed that the initial spine sizes distribution of the original 3 spine and the "15 spine- 3 range" groups were not significantly different (Kolmogorov-Smirnov two-sample test led to a p-value of 0.718). **d)** We compared the temporal plasticity dynamics of the 15 spine and "15 spine - 3 range" experiments and found no significant differences (albeit a slightly bigger growth in the trimmed set) between the two conditions. N values can be found in Table 1.
